# High fat diet-induced changes of mouse hepatic transcription and enhancer activity can be reversed by subsequent weight loss

**DOI:** 10.1038/srep40220

**Published:** 2017-01-10

**Authors:** Majken Siersbæk, Lyuba Varticovski, Shutong Yang, Songjoon Baek, Ronni Nielsen, Susanne Mandrup, Gordon L. Hager, Jay H. Chung, Lars Grøntved

**Affiliations:** 1 Department of Biochemistry and Molecular Biology, University of Southern Denmark, 5230 Odense M, Denmark; 2 Laboratory of Receptor Biology and Gene Expression, Center for Cancer Research, National Cancer Institute, National Institute of Health (NIH), Bethesda, MD 20892, USA; 3 Laboratory of Obesity and Aging Research, Genetics and Developmental Biology Center, National Heart Lung and Blood Institute, NIH, Bethesda, MD 20892, USA

## Abstract

Epigenetic factors have been suggested to play an important role in metabolic memory by trapping and maintaining initial metabolic changes within the transcriptional regulatory machinery. In this study we fed mice a high fat diet (HFD) for seven weeks followed by additional five weeks of chow, to identify HFD-mediated changes to the hepatic transcriptional program that may persist after weight loss. Mice fed a HFD displayed increased fasting insulin levels, hepatosteatosis and major changes in hepatic gene transcription associated with modulation of H3K27Ac at enhancers, but no significant changes in chromatin accessibility, indicating that HFD-regulated gene transcription is primarily controlled by modulating the activity of pre-established enhancers. After return to the same body weight as chow fed control mice, the fasting insulin, glucose, and hepatic triglyceride levels were fully restored to normal levels. Moreover, HFD-regulated H3K27Ac and mRNA levels returned to similar levels as control mice. These data demonstrates that the transcription regulatory landscape in the liver induced by HFD is highly dynamic and can be reversed by weight loss. This provides hope for efficient treatment of early obesity-associated changes to hepatic complications by simple weight loss intervention without persistent reprograming of the liver transcriptome.

Diet-induced obesity (DIO) is associated with metabolic changes that significantly increase the risk of cardiovascular complications, cancer and diabetes. Recent reports suggest that by 2025 a fifth of the world’s population will be obese^1^. Significant resources are invested in treatment strategies of complications associated with obesity. Since obesity arises as a complex interaction between inherited traits and the environment, life style intervention strategies, such as exercise and change of diet, are not necessarily obvious. Moreover, despite successful control of metabolic dysfunction, such as type 2 diabetes, the remaining metabolic memory leads to increased risk of metabolic diseases^2^,^3^,^4^. Recent studies have suggested that epigenetic factors may contribute to the metabolic memory in liver tissue^5^,^6^, indicating that efforts to identify and modify these factors could be beneficial for metabolic intervention and help prevent relapse after treatment.

Epigenetic factors, such as DNA methylation and histone modifications, are associated with transcription factor (TF) accessibility to chromatin, enhancer activity and ultimately regulation of gene expression^7^,^8^,^9^,^10^. Specific chromatin remodeling and accessibility in these enhancers are manifested in probabilistic transcriptional changes of connected genes. A number of techniques, including DNase-, ATAC- and FAIRE-seq, are available to probe changes in chromatin accessibility^11^. Importantly, chromatin accessibility to DNase correlates with TF occupancy, emphasizing that this method efficiently identifies regulatory regions genome-wide^8^,^10^,^12^. However, information from genome-wide chromatin accessibility by itself does not provide sufficient information of the activity states in identified regulatory regions. Thus, parallel detection of enhancer activity can be obtained by ChIP-seq targeting H3K27Ac and/or MED1, or by quantification of enhancer RNA expression by GRO-seq^12^,^13^,^14^,^15^.

Consumption of HFD for several weeks leads to DIO and is associated with hepatosteatosis in laboratory animal models, such as C57BL/6 mice. This process is controlled by a range of molecular mechanisms, including change of the hepatic transcriptional program^5^,^16^,^17^. Hepatosteatosis is reversible^18^, yet it has been suggested that DIO in rodents followed by weight loss leaves persistent changes in hepatic chromatin organization (probed by FAIRE-seq) and persistent pattern of gene expression^6^. In agreement with these studies, it has been reported that hepatic DNA methylation in humans is changed by obesity, and weight loss by bariatric surgery does not fully reverse obesity-associated DNA methylation^19^. In contrast, other studies have reported complete reversal of HFD-induced changes of metabolism, hepatic circadian gene transcription and circadian behavior^20^,^21^. Here we have used an integrated genomics approach to carefully assess whether transcription and enhancer activity regulated by HFD are reversible. This included profiling of the transcriptome by RNA-seq, chromatin accessibility by DNase-seq and enhancer activity by H3K27Ac ChIP-seq. We show that HFD-induced hepatosteatosis is fully reversible at the macroscopic level as well as at the genomic level.

## Results

### HFD-induced hyperinsulinemia and increased hepatic lipid accumulation are reversed by weight loss

**Figure 1:**
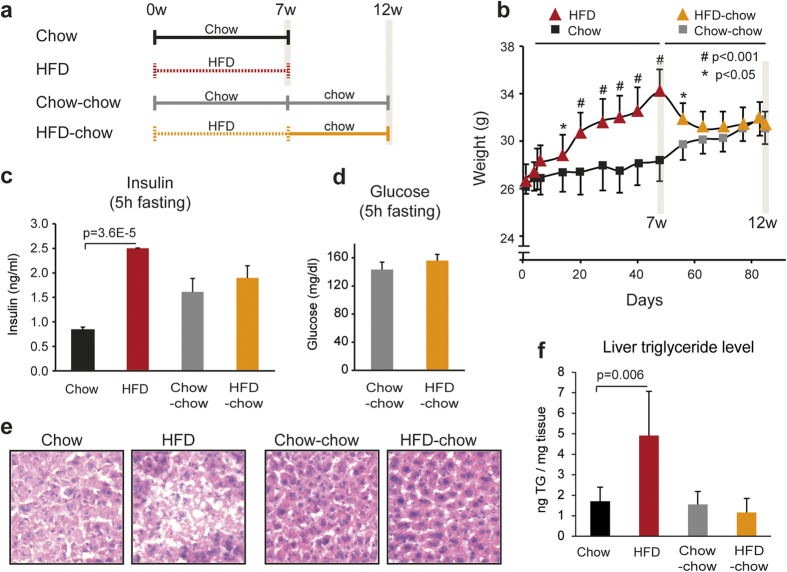
HFD-induced hyperinsulinemia and increased hepatic lipid accumulation is reversed by return to chow diet. (**a)** Male 12 week old C57BL/6 J mice were fed a HFD (broken line, red) or chow diet (solid line, black) for seven weeks (7w) where half of the mice were sacrificed (Chow and HFD). The remaining HFD and chow mice were switched to or continued on a chow diet for additional five weeks (12w), respectively (Chow-chow, solid gray line and HFD-chow, broken orange line). (**b)** Mouse body weight was examined throughout the experiment (n > 4). (**c)** Serum glucose levels (HFD-chow and Chow-chow n = 6) and (**d)** insulin levels (chow and HFD n = 3, Chow-chow and HFD-chow n = 6) after 5 h of fasting. (**e)** Representative H&E staining of liver sections (8 μm, 20x magnification) and **f)** quantification of liver TG levels per tissue weight (mg) in Chow, HFD, Chow-chow and HFD-chow mice (n = 3). Statistical test was performed by two-tailed t-test. Error bars represent standard error of the mean (SEM).

To investigate the effects of HFD followed by weight loss, the following experimental setup was used: two groups of 12 week old male C57BL/6 J mice were either fed a HFD or a chow diet ad libitum for seven weeks and subsequently half of the mice from each group where sacrificed and livers were isolated (Fig. 1a, termed HFD and Chow, respectively). The remaining half of the HFD and Chow mice were switched to or continued on a chow diet, respectively, for additional five weeks after which livers were isolated. Mice receiving chow for the entire period of the experiment are termed Chow-chow and mice initially fed HFD for seven weeks and then shifted to chow for five weeks are termed HFD-chow (Fig. 1a). HFD mice displayed significant weight gain (Fig. 1b), hyperinsulinemia (Fig. 1c) and triglyceride (TG) accumulation in the liver as compared to chow fed control mice (Fig. 1e,f). Following weight loss of the HFD-chow group (Fig. 1b), serum insulin levels returned to normal (Fig. 1c) and no difference in serum glucose levels compared to Chow-chow mice was observed (Fig. 1d). Moreover, liver morphology and the accumulated TG levels observed in the HFD group returned to similar levels in HFD-chow as in Chow-chow (Fig. 1e,f).

### The liver transcriptome regulated by HFD

**Figure 2:**
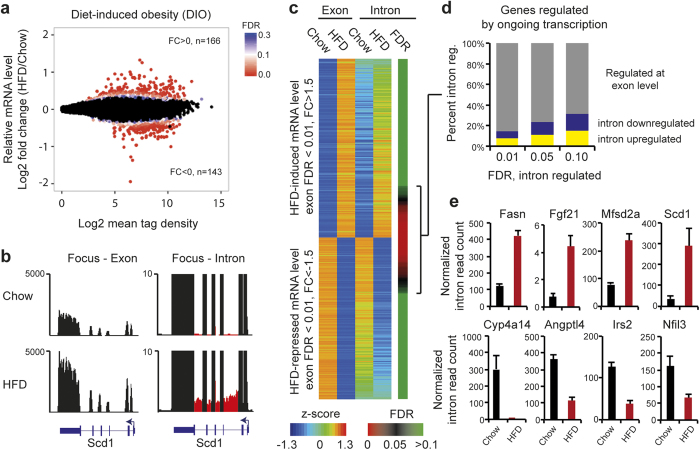
The liver transcriptome of HFD mice before and after weight loss. RNA-seq from livers of three mice from each group; Chow, HFD was analyzed for (**a)** differential mRNA levels using DESeq2^22^. FDR value < 0.3 is indicated with heatmap. Data points with FDR > 0.3 are colored black. The number of genes with log2 fold change (FC) more or less than zero and FDR < 0.05 is indicated in figure. (**b)** RNA-seq data visualization of Scd1 locus in Chow and HFD mice with focus on exon (left) and intron (right) RNA reads. Intronic reads are marked red. (**c)** Quantification of exon versus intron mRNA reads using the iRNA pipeline^23^ in Chow and HFD mice, grouped into HFD-induced or -repressed genes. Regulated genes are scored based on FDR (exon) <0.01 and 1.5 fold change (FC). A z-score is calculated for the RNA-seq data to normalize tag counts within exons and introns. FDR values from HFD-regulated intron RNA expression are indicated with a heatmap. (**d)** Fraction of genes up- (yellow) and downregulated (blue) by ongoing transcription after HFD at different FDR for HFD-regulated intron RNA expression. (**e)** Normalized (Reads Per Kilobase Million) intron read count from HFD-regulated genes: Scd1, Fasn, Fgf21, Mfsd2a (upregulated by HFD, upper panel) and Cyp4a14, Angptl4, Irs2, and Nfil2 (downregulated by HFD, lower panel). Error bars represent SEM.

To investigate the genome-wide changes of RNA levels in the liver induced by HFD and to compare those to livers from mice that underwent subsequent weight loss, we performed RNA-seq from livers of three mice in each of the four groups: HFD, Chow, HFD-chow, Chow-chow. The libraries were sequenced to a depth of at least 7–11 million uniquely aligned tags (Supplementary Table S1) and analyzed for differential mRNA levels using DESeq2^22^. DIO resulted in a dramatic change in the liver transcriptome (Fig. 2a). More than 160 genes showed a significant increase and 140 genes had a decrease in mRNA levels when compared to chow-fed mice at FDR < 0.05 when corrected for multiple testing (Fig. 2a). GO term analysis suggested that regulated mRNAs are involved in metabolic processes such as lipid, fatty acid and cholesterol metabolism (Supplementary Table S2).

To obtain a more precise readout of ongoing gene transcription, we prepared RNA-seq libraries from total RNA depleted for ribosomal RNA and used random hexamer primers for cDNA synthesis. This allowed us to quantify exon as well as intron RNA genome-wide. Intronic RNA reads were specifically used to measure nascent RNA expression^23^. For example, the mRNA level of stearoyl-Coenzyme A desaturase 1 (Scd1), a gene directly involved in fatty acid metabolism, was differentially regulated by HFD both at the levels of exon and intron RNA reads, indicating that Scd1 was regulated by ongoing transcription of the gene in response to HFD (Fig. 2b). To identify genome-wide changes of nascent RNA, we used the iRNA pipeline for specific quantification of exonic versus intronic RNA reads^23^. Genome-wide analysis demonstrated differential intronic RNA levels in 15–32% of the exome-regulated mRNAs at FDR between 0.01–0.1 when corrected for multiple testing (Fig. 2c,d). This demonstrates significant nascent RNA synthesis from a subset of HFD-regulated genes at the time of analysis, yet the vast majority of differential mRNA expression measured after several weeks of HFD exposure could not be assigned to ongoing gene transcription at the time of measurement. Importantly, differential nascent RNA synthesis was measured from a number of genes known to be involved in DIO, such as upregulation of fatty acid synthase (Fasn), fibroblast growth factor 21 (Fgf21), major facilitator superfamily domain containing 2A (Mfsd2a) and Scd1, and downregulation of cytochrome P450, family 4, subfamily a, polypeptide 14 (Cyp4a14), angiopoietin-like 4 (Angtpl4), insulin receptor substrate 2 (Irs2) and nuclear factor, interleukin 3 regulated (Nfil3) (Fig. 2e). Pathway analysis of intron-regulated genes using the curated reactome database^24^,^25^ suggested that ongoing HFD-regulated gene expression is controlled by transcriptional networks regulated by sterol-regulatory element binding protein- (SREBP), carbohydrate-responsive element-binding protein (ChREBP) and peroxisome proliferator activated receptor (PPAR) (Supplementary Table S3).

### Chromatin accessibility remains largely unchanged in response to HFD

**Figure 3:**
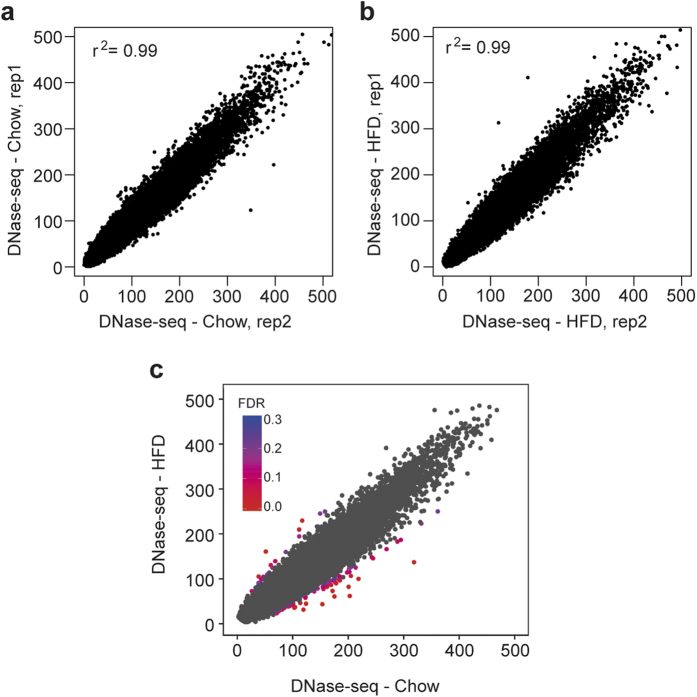
Chromatin remodeling remains largely unchanged in response to HFD. Scatter plots illustrating level of DNase accessibility for replicate 1 and 2 of (**a)** Chow and (**b)** HFD and (**c)** in HFD versus Chow group. Differential DNase accessibility is indicated by FDR values identified by DESeq2 (n = 2). FDR levels are shown with indicated heatmap, where red represents the most statistical significant data points. Gray data points represents differential DNase accessibility at FDR > 0.3.

To determine whether HFD-mediated changes of transcription are associated with changes in chromatin accessibility, we performed DNase-seq on livers from HFD and Chow mice. Isolated nuclei from liver tissue of two Chow and HFD mice were treated with 40 U or 60 U of DNase I. DNA fragments less than 500 bp were purified using ultra centrifugation and sequenced. Sequenced libraries from 40 U and 60 U DNase were combined for each biological replicate and a total of more than 74,000 DNase accessible regions were identified. Correlative analysis suggested little variability of DNase-seq accessibility among biological replicates (Fig. 3a,b). Interestingly, we detected very few significant changes in chromatin accessibility following HFD (Fig. 3c), suggesting that the transcriptional changes in DIO are regulated predominantly by changes in pre-established regulatory regions and not by *de novo* remodeling of chromatin.

### Changes of H3K27Ac levels in pre-established enhancers following HFD

**Figure 4:**
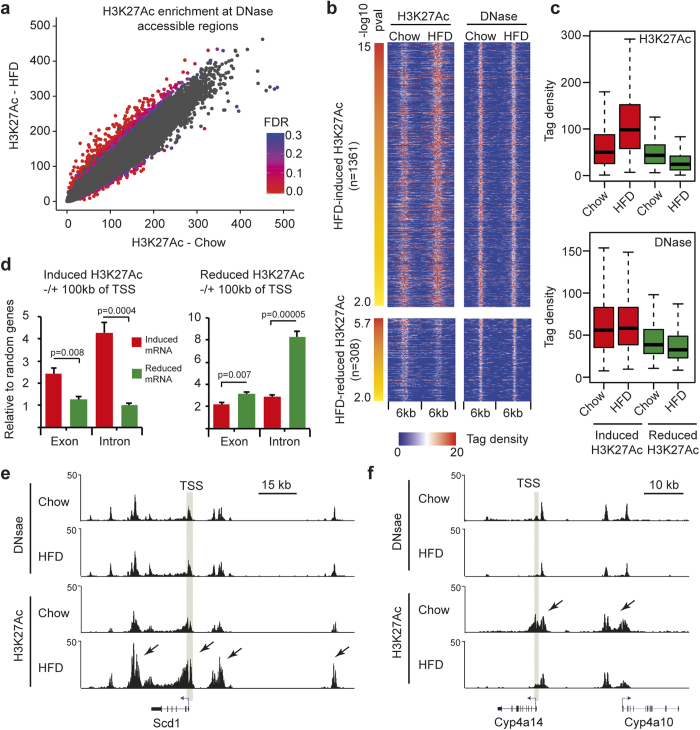
Pre-established enhancers change H3K27Ac level in response to HFD. (**a)** H3K27Ac ChIP-seq enrichment was quantified at all identified DNase accessible regions and DESeq2 was used to identify differential H3K27Ac. FDR is indicated with heatmap, where red represents the most statistical significant data points. Gray data points represents differential H3K27Ac at FDR > 0.3. (**b)** Differentially H3K27Ac (p < 0.01) ranked according to p-value. Corresponding DNase accessibility is plotted in the left panel. (**c)** H3K27Ac ChIP-seq and DNase-seq tag densities at regions where HFD induce (red) and reduce (green) H3K27Ac. (**d)** Enrichment of induced (left panel) or reduced (right panel) H3K27Ac relative to multiple rounds (n = 4) of 200 randomly selected genes within 100 kb of TSS of genes HFD up- (red) or downregulated (green) genes scored by intron or exon reads. Statistical test was performed by two-tailed t-test. Error bars represent SEM. (**e)** Screen shots of DNase accessibility and H3K27Ac regions around Scd1 and (**f)** Cyp4a14 genes. TSS: Transcription start site. Arrows indicate differentially regulated H3K27Ac.

Enhancer activity correlates positively with the level of H3K27 acetylation^13^,^26^. Therefore, to map modifications in enhancer activity in liver tissue in response to HFD we profiled H3K27Ac by ChIP-seq using three biological replicates from each group of mice. H3K27Ac enrichment was quantified at all identified DNase accessible regions and DESeq2 was used to determine differential acetylation levels. We identified a total of 1,669 DNase accessible regions that changed H3K27Ac level in response to HFD at a p-value < 0.01. Corrected for multiple testing and FDR < 0.1 reduced this number to 883. The majority of these regions showed an increase in histone acetylation (Fig. 4a,b). In agreement with genome-wide DNase accessibility analysis (Fig. 3c), little change in DNase accessibility (Fig. 4c, lower panel) was observed in the differential H3K27-acetylated regions (Fig. 4c, upper panel). To correlate HFD-mediated changes of H3K27Ac with gene expression, we analyzed enrichment of differentially regulated H3K27Ac within 100 kb of HFD-regulated genes (intron- and exon-regulated) relative to sets of 200 randomly selected genes. We observed that HFD-induced genes, both at the intron and exon level, are enriched for increased H3K27Ac in nearby regions. In contrast, HFD-repressed gene expression correlated with nearby reduced H3K27Ac (Fig. 4d), which is specifically evident for genes regulated at the level of ongoing transcription (Fig. 4d, intron reads). For example, the HFD-induced gene Scd1 (Fig. 2b) is flanked by four HFD-induced enhancers positioned >10 kb from the transcriptional start site (TSS) (Fig. 4e). Likewise, the HFD-repressed gene, Cyp4a14, is flanked by regions of repressed H3K27Ac in HFD mice (Fig. 4f). Furthermore, in agreement with a larger proportion of HFD-induced acetylation at H3K27 sites, we observed a higher percentage of HFD-induced genes with nearby enrichment of H3K27Ac as compared to repressed genes (Supplementary Figure S4).

To gain insights into candidate TFs that may be involved in regulation of enhancer activity by HFD, we extracted all DNase accessible regions that were differentially regulated at H3K27Ac in response to HFD (at p < 0.01) and submitted these for enriched motif analysis using DNA motifs for TFs curated by HOMER^27^. Since a large percentage of activated genes harbors nearby regions with increased H3K27Ac, we focused on regions that contained increased H3K27Ac. The ten most enriched motifs identified were binding motifs for PPAR, CCAAT-enhancer-binding protein (C/EBP), Hepatocyte nuclear factor 4 (HNF4) and SREBP (Supplementary Figure S5a). These possible regulators were also in agreement with the reactome pathway analysis (Supplementary Table S3). Quantification of the motif strength within the most enriched motifs at regions with either induced or reduced H3K27Ac or randomly selected DNase accessible regions showed that C/EBP, SREBP, NF-Y, ATF4 and ATF1 motifs were significantly enriched at regions with increased H3K27Ac (Supplementary S5b). The C/EBPα, SREBP and NF-Y transcription factors have previously been shown to regulate expression of genes involved in lipogenesis^28^,^29^,^30^,^31^. Moreover, ATF4 depletion has been shown to protect mice from diet-induced hepatosteatosis^32^,^33^.

### Weight loss reverses the HFD-regulated hepatic transcriptome

**Figure 5:**
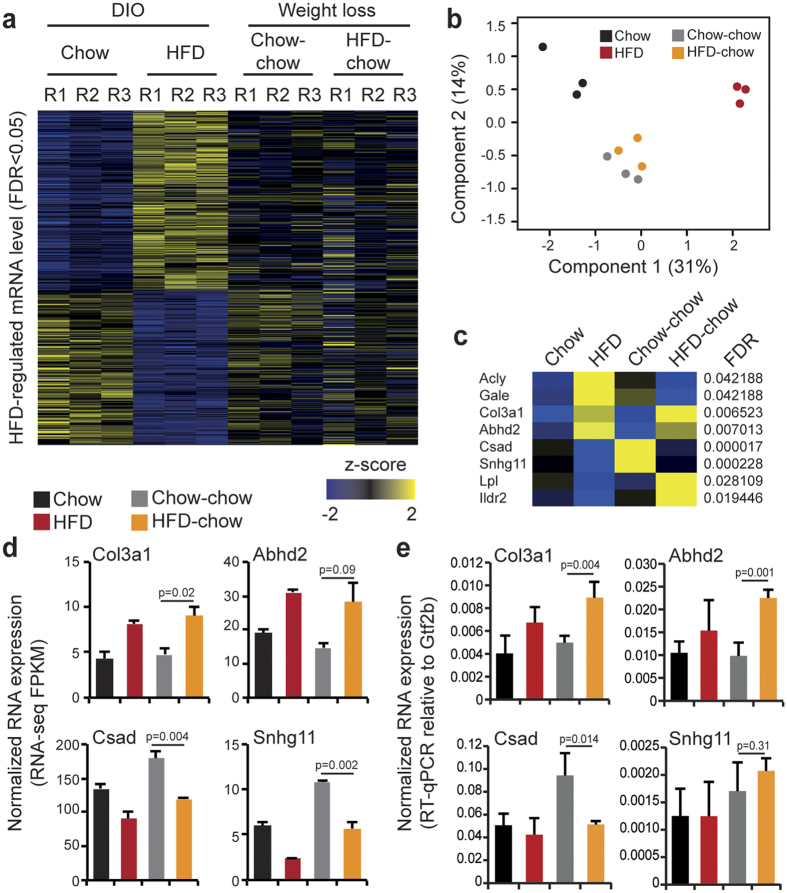
HFD-regulated transcriptome returns to normal after weight loss. (**a)** RNA-seq read count for each biological replicate in Chow, HFD, Chow-chow and HFD-chow conditions at HFD-regulated genes (at FDR < 0.05). Read counts are visualized as z-scores. (**b)** Principle component analysis of each biological RNA-seq replicate in Chow, HFD, Chow-chow and HFD-chow conditions. (**c)** RNA-seq read count at HFD-regulated genes also regulated after weight loss (Genes with FDR < 0.05 is visualized). Average read counts (n = 3) are visualized as z-scores. (**d)** Normalized mRNA expression from selected HFD-regulated genes persistently expressed after weight loss. RNA-seq tag count at exons is plotted as Fragments Per Kilobase per Million mapped reads (FPKM). Statistical test was performed by two-tailed t-test, n = 3. Error bars represent SEM. (**e)** RT-qPCR validation RNA-seq data. Data is normalized to General transcription factor 2b (Gtf2b) expression. Statistical test was performed by two-tailed t-test, n = 3–4. Error bars represent SEM.

Replacing HFD with chow for five weeks (HFD-chow mice) normalized body weight and reversed the hepatosteatosis observed in HFD mice (Fig. 1b,f). To measure effects on the transcriptome, we performed RNA-seq analysis on three livers from Chow-chow and HFD-chow mice (Supplementary Table S1) with initial focus on HFD-regulated genes (Fig. 5a). Principal component analysis of replicate RNA-seq data from HFD-regulated genes indicated small variability between individual mice and none of the chow-containing feeding regimens clustered together with HFD mice (Fig. 5b), which was supported by pairwise Pearson correlation analysis between replicate RNA-seq data (Supplementary Figure S6a). In addition, principle component analysis and pairwise Pearson correlation analysis indicated that HFD-chow RNA-seq data clustered together with Chow-chow data, suggesting an overall normalization of the HFD-regulated hepatic transcriptome following weight normalization. Statistical analysis using DESeq2 only identified eight HFD-regulated mRNA’s at FDR < 0.05 (corrected for multiple testing) that were differentially regulated in HFD-chow as compared to control (Chow-chow) livers (Fig. 5c). Only four of these mRNA’s were persistently up- or down regulated (Fig. 5d), indicating that only a very small subset of HFD-regulated genes were not reversed to the levels observed in Chow-chow mice following weight loss. Validation of expression by RT-qPCR confirmed similar expression profiles for three out of the four genes (Fig. 5e), including collagen alpha1 (III) (Col3a1). Col3a1 has been linked to hepatic fibrosis together with genes such as alpha-smooth muscle actin (Acta2), transforming growth factor-β1 (Tgfb1), desmin (Des) and collagen alpha1 (I) (Col1a1)^34^. None of these additional fibrosis-associated genes showed persistent expression after weight loss of HFD-chow mice (Supplementary Figure S6b). This indicates that fibrosis is not pronounced in the liver after weight loss. Interestingly, when all annotated genes were included in the differential gene expression analysis of HFD-chow versus Chow-chow mice, we identified a number of significantly (FDR < 0.05, corrected for multiple testing) regulated genes (Supplementary Figure S6c). This group represents genes that were specifically changed upon weight loss. Accordingly, only a small fraction of these genes overlaps with HFD-regulated genes (Supplementary Figure S6d).

### HFD-regulated H3K27Ac levels return to normal after weight loss

**Figure 6:**
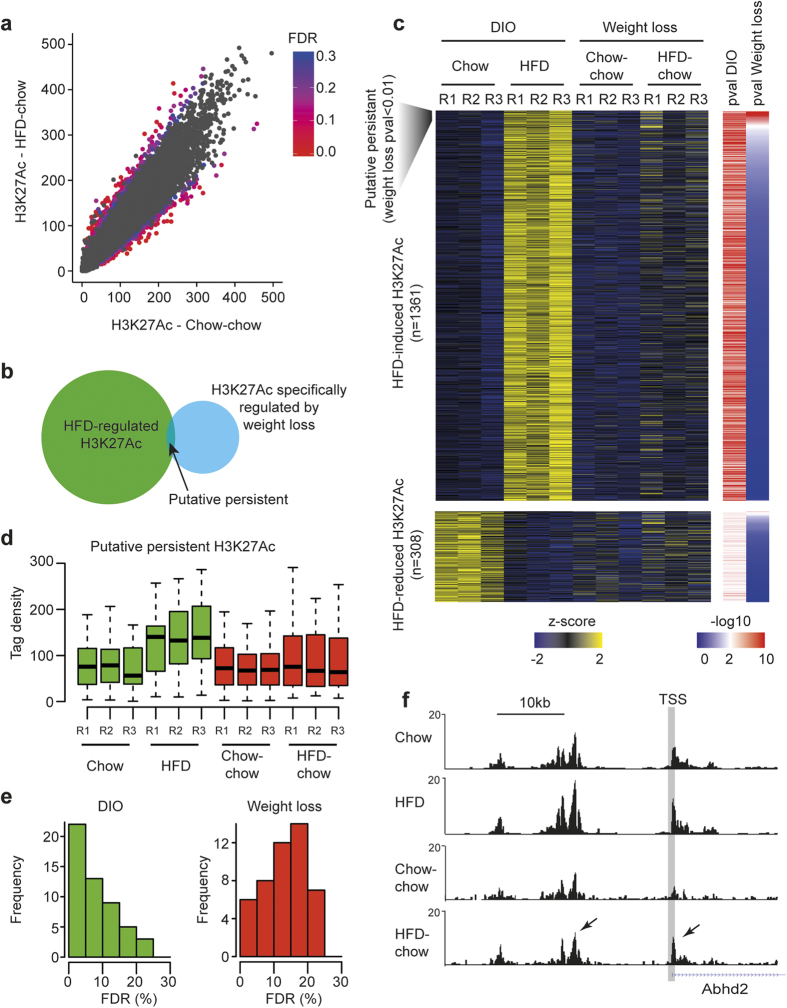
HFD-regulated H3K27Ac levels returns to normal after weight loss. (**a)** H3K27Ac ChIP-seq in livers from HFD-chow and Chow-chow mice. Significance levels (FDR) are shown with color codes (right panel), where red represents most statistical significant values. Gray data points represents differential H3K27Ac at FDR > 0.3. (**b)** Venn diagram of HFD-regulated H3K27Ac regions at FDR < 0.1 (green) overlapping with differentially regulated H3K27Ac regions in HFD-chow mice compared to Chow-chow at FDR < 0.1 (blue). Overlapping regions represent putative persistent H3K27Ac deposited by HFD. (**c)** H3K27Ac level in DIO mice (HFD group) compared to DIO after weight loss (HFD-chow group). All HFD-regulated enhancers are shown. H3K27Ac level is normalized to a Z-score for each individual replicate. Heatmaps representing p-values (calculated by DESeq2) for HFD-regulated H3K27Ac is shown to the right. Red color represents most statistical significant changes. Putative persistent regions are identified using a cutoff of p-value < 0.01. (**d)** Tag densities of putative persistent H3K27Ac in three replicates from Chow, HFD, Chow-chow and HFD-chow mice. Green boxplots shows H3K27Ac changes at putative persistent acetylated enhancers in Chow and HFD fed mice. Red boxplots shows H3K27Ac changes at putative persistent acetylated enhancers in Chow-chow and HFD-chow fed mice. (**e)** FDR of differentially putative persistent H3K27Ac regions in Chow compared to HFD (green, DIO, left panel) and Chow-chow compared to HFD-chow (red, weight loss, right panel). (**f)** H3K27Ac in Chow, HFD, Chow-chow, and HFD-chow mice around TSS of the Abhd2 gene. Arrows mark regions with changed H3K27Ac after weight loss.

It has previously been suggested that HFD-induced chromatin remodeling (probed by FAIRE-seq) in the mouse liver genome persists after weight loss^6^. To investigate whether HFD-induced H3K27Ac changes are maintained after weight loss, we profiled H3K27Ac in three livers from HFD-chow mice and compared those to Chow-chow mice. Principle component analysis and pairwise Pearson correlation analysis (Supplementary Figure S7) indicated that H3K27Ac at HFD-regulated enhancers was similar between HFD-chow and Chow-chow livers, suggesting that HFD-regulated H3K27Ac returns to normal after weight loss. We identified 1,324 DNAse accessible regions showing differentially regulated H3K27Ac at p-value < 0.01 and reduced these to 254 when corrected for multiple testing at FDR < 0.1 (using DESEq2). The vast majority of these H3K27Ac regions in HFD-chow mice did not overlap with those in HFD mice (Fig. 6b) with only a minor common H3K27-acetylated fraction that remained after weight loss at p < 0.01 (Fig. 6c). Additional analysis showed that the small fraction of putative persistent H3K27Ac was not significantly acetylated after weight loss in HFD-chow mice (Fig. 6d), and when corrected for multiple testing revealed that the vast majority of differential H3K27Ac at these regions had a relatively high FDR, as compared to H3K27Ac regulated by DIO (Fig. 6e). These data indicate that most putative persistent H3K27Ac sites likely represent false positives. Thus, we conclude that overall H3K27Ac changes induced by HFD did not persist after weight loss, which agrees with the RNA expression data presented in Fig. 5. Interestingly, an enhancer near Abhydrolase Domain Containing 2 (Abhd2) was persistently H3K27-acetylated after weight loss (Fig. 6f), which agrees with RNA expression data (Fig. 5d). We observed that differentially H3K27Ac-regulated regions after weight loss did not overlap with HFD-regulated H3K27Ac (Fig. 6b). In contrast, these regions represent enhancers that are regulated as a consequence of weight loss.

## Discussion

Liver dysfunction is linked to pathophysiology of obesity, where hepatosteatosis can lead to insulin resistance, type II diabetes^35^, fibrosis and even cirrhosis^36^,^37^. Under normal physiological conditions, the liver handles synthesis, storage, and redistribution of lipids, amino acids and glucose in a highly coordinated and dynamic manner that is regulated by food intake, environmental, circadian, hormonal, and neuronal stimuli^38^,^39^. These require enormous hepatic transcriptional plasticity through coordinated crosstalk between the environment and the epigenome, orchestrated by chromatin remodeling enzymes and DNA-bound TFs and their co-factors^40^. When mammals are challenged with HFD for several weeks, it requires significant re-programming of hepatic gene transcription to handle excess nutrients. These include adaptive regulation of genes involved in glucose and lipid metabolism^41^, TG accumulation in the liver and disruption of liver circadian clock^17^,^20^, which agrees with HFD-regulated pathways identified in this study (Supplementary Tables S2 and S3). It has been shown that a shift from HFD to regular chow reverses the dysregulated circadian clock at a transcriptional^20^ as well as at a behavioral level^21^, suggesting that the change of diet can reverse HFD-provoked deregulation of hepatic gene transcription and ultimately normalize lipid and glucose metabolism^42^. However, a recent study has suggested that significant changes of chromatin organization and gene transcription in the liver may persist after a shift from HFD to regular chow diet in DIO mice^6^. However, that study did not achieve a complete normalization of the weight and hepatic TG accumulation after diet reversal^6^. Thus, it is possible that observed persistent remodeled chromatin initiated by HFD may be a consequence of incomplete weight normalization and residual TG accumulation in the liver.

In the experimental setup used in the study presented here, mice were fed HFD for seven weeks followed by a change to normal chow until they lost the weight they had gained (five weeks), reversed serum hyperinsulinemia and TG accumulation in the liver. As expected, HFD regimen resulted in DIO and dramatically changed the liver transcriptome. By applying the iRNA-seq pipeline to RNA-seq analysis we identified ongoing transcriptional changes promoted by HFD. Interestingly, less than a quarter of the HFD-regulated transcriptome was actively transcribed when compared to chow-fed control mice, implying considerable regulation at the level of mRNA turnover. Importantly, ongoing transcription regulated by HFD specifically correlated with nearby changes in H3K27Ac at DNase accessible regions. In contrast, DNase accessibility itself was not substantially modified by HFD, suggesting that HFD does not lead to establishment of new enhancers. Rather, the activity of already established enhancers is differentially regulated by the diet. Pathway analysis of differentially expressed genes and DNA motif analysis of these enhancers suggested that HFD induces SREBP-, ATF-4- and C/EBP-regulated transcriptional networks, in agreement with previous reports^43^,^44^,^45^,^46^. Thus, the experimental HFD setup used in our study resulted in hyperinsulinemia and hepatosteatosis associated with specific changes in enhancer activity and gene transcription likely regulated by transcription factors such as SREBP, ATF-4 and C/EBP.

After seven weeks of HFD, mice were switched to chow diet (HFD-chow) and weight changes were carefully monitored and compared to control mice of the same age. A significant weight loss was observed within a week on chow diet. After two weeks, there were no statistically significant differences in weight between HFD-chow and Chow-chow groups. We housed both groups of mice for an additional three weeks on chow diet to maintain matched weight before final analysis. Notably, a similar weight loss pattern has been observed in some diet reversal studies^21^ but not others^6^. In the study by Leung *et al*., reporting persistent chromatin structural and transcriptional changes in DIO mice after weight loss, no significant weight loss was observed. Instead, HFD mice stopped gaining weight when returned to chow diet^6^. This study used the same C57BL/6 J mouse strain, thus these marked differences may be a consequence of the experimental setup including differences in HFD formula used, age of mice at the beginning of treatment, and/or housing facility. For example the diet used in the study by Leung *et al*. (diet formula D12266B) has an overall fat and carbohydrate composition of 32% kcal and 51% kcal, respectively, whereas the diet used in this study (diet formula D12327) has a 40% kcal carbohydrate and 40% kcal fat composition. Interestingly, equal amounts of saturated fatty acids (SFA) and monounsaturated fatty acids (MUFA) are present in D12266B, whereas D12327 primarily contains SFA. It has previously been shown that administration of diet rich in SFA to rodents’ leads to less dramatic change of insulin sensitivity and TG accumulation compared to a mixed SFA-MUFA diet^47^. This implies that the degree of insulin resistance and hepatosteatosis may impact the effect of diet reversal. Indeed, the degree of NAFLD has an effect on the beneficial hepatic recovery after bariatric surgery in humans^48^.

When we compared our data to those reported by Leung *et al*.^6^ we noticed another important difference in the study design applied to identify persistent HFD-induced changes of the liver transcriptome after reversed diet. Leung *et al*. used a study design where three groups of mice were followed for 16 weeks in total. One group received chow, a second HFD and a third HFD for 8 weeks followed by chow for 8 weeks. They performed liver RNA-seq and FAIRE-seq after 16 weeks for all three groups of mice to discover differential gene expression and chromatin accessibility. This study design allowed Leung *et al*. to score transcriptional differences in the liver between the different feeding regiments at the end point of the study. However, this design did not allow Leung *et al*. to identify actual affects of HFD before the mice returned to chow. To correct for this, the study used previously published FAIRE-seq data from HFD fed mice (8 weeks of HFD)^5^, although without additional statistical analysis. Moreover the study did not use any reference point to HFD-induced changes preceding diet reversal for the transcriptomic analysis. Thus, without a HFD reference point for statistical analysis, the subsequent analysis may as well score changes that originate from mice placed on normal diet after being obese.

In the study presented here we used a reference point of obese mice before diet reversal. This allowed us to statistically score differential gene transcription and enhancer activity that originates specifically from HFD, and observe if these HFD-induced changes are expressed after diet reversal. We observed that weight loss after HFD resulted in almost complete reversal of the HFD-regulated transcriptome and H3K27 acetylation level. Very few HFD-regulated genes, including Col3a1 and Abhd2, did not return to control levels after weight loss. Col3a1 expression in hepatic stellate cells is associated with liver fibrosis^49^. We investigated the expression of other genes linked to hepatic fibrosis and found no evidence of persistent expression of fibrosis-associated genes. These results suggest that initial HFD-mediated hepatic fibrosis may be reversed by diet-induced weight loss. In agreement with our conclusions, a number of studies have indicated that early onset fibrosis is significantly reduced after weight loss in obese subjects^50^. Abhd2 has been characterized as triacylglycerol (TAG) lipase and ester hydrolase^51^. Disruption of Abhd2 in mice leads to a decrease in alveolar type II cells in the lung and accumulation of macrophages in the lungs^52^. Moreover, Abhd2 has been linked to vascular smooth muscle cells (SMC) migration^53^ but so far no apparent function in liver has been reported. Since Abhd2 is a lipase, it may be involved in mobilization of TGs in a steatotic liver in response to diet-induced weight loss. The potential role of Abhd2 in DIO warrants further investigation.

The data presented in this work clearly shows that normalization of hepatic TG accumulation induced by HFD and subsequent return to normal body weight normalizes DIO-regulated hepatic transcription and enhancer activity. Thus, reversed hepatosteatosis by diet-mediated weight loss is accompanied by normalized transcriptional regulation, illustrating a highly dynamic hepatic transcriptome regulated by diet.

## Materials and Methods

### Animal work

All experiments were approved by the ACUC (Animal Care and Use Committee) of the NHLBI, NIH and all methods were performed according to relevant guidelines and regulations from ACUC. C57BL/6J mice (Jackson Laboratory) were fed either a HFD (D12327, Research Diet, 40% calories from fat (of which 78% are SFA, 7% MUFAs and 15% polyunsaturated fatty acids), 40% calories from carbohydrate and 20% calories from protein or chow diet (NIH-31, Zeigler Brothers Inc., 8% calories from fat) for seven weeks from 12 weeks of age (groups termed HFD and Chow, respectively). After seven weeks, half of the HFD and Chow mice where sacrificed by CO_2_ narcosis followed by cervical dislocation, livers were dissected and processed for RNA purification, nuclei isolation or frozen in liquid nitrogen for subsequent studies. Blood samples were taken as indicated and processed for metabolic measurements. The other half of the HFD and Chow mice where switched to or continued on a chow diet, respectively, (termed HFD-chow and Chow-chow) for an additional five weeks. See Fig. 1a for experimental setup. Body weights were measured once a week.

### Blood glucose measurement

Blood sample was obtained by nicking the tail vein with a sterile scalpel blade. One droplet of blood was placed on a glucose test strip and read with a glucometer (Ascensia).

### Serum insulin measurement

To measure serum, whole blood, directly drawn from the tail vein, was put into a tube with no anti-coagulant. Blood clot was allowed to form at room temperature for 30 min. The clotted blood was then centrifuged at 4000 rpm for 15 minutes at 4 °c. Serum insulin level was measured using an ELISA kit (EMD Millipore).

### Hematoxylin and Eosin (H&E) staining and TG measurements

Frozen liver sections were embedded in tissue freezing medium (Leica, 4020108926), cut in 8 μm sections, and stained with Mayers Hematoxylin (Sigma, MHS16) and aqous Eosin Y solution (Sigma, HT110216), dehydrated with ethanol and TissueClear (Sakura, #1466). For TG measurements, ~50 mg of tissue were homogenized in 500 ul isopropanol using UltraThorax and subsequently sonicated (QSonica) 2 × 5 sec at 25% amplitude. To measure initial free glycerol in samples, 2 μl sample supernatant and glycerol standards were mixed with 100 μl free glycerol reagent (Sigma, F6428) in duplicates, incubated for 5 min at RT followed by OD measured at 540 nm (Initial absorbance (IA)). Subsequently, 25 ul of triglyceride reagent containing lipoprotein lipase (Sigma, T2449) was added and incubated for 30 min at 37 °C, followed by glycerol measurement at OD 540 nm (Final absorbance (FA)). For calculation of TG content, IA was subtracted from FA values, corrected according to manufacturer’s instructions and divided by tissue weight (mg).

### RNA isolation and Illumina sequencing

Approximately 5 mg of liver tissue were homogenized using UltraThorax and RNA was purified using TRIzol-RNA lysis reagent (ThermoFisher) according to manufacturer’s instructions. RNA quality was assessed using the Agilend 2100 Bioanalyzer or the Fragment Analyzer (AATI). Total RNA (1 μg) was either depleted for ribosomal RNA using Ribo-Zero Magnetic Kit (Epicentre) followed by cDNA synthesis using random primers or prepared for sequencing using polydT-mediated cDNA synthesis, both procedures according to manufacturer’s (Illumina) instructions. Subsequent library preparation was performed using TruSeq RNA sample prep kit (Illumina). Library quality was assessed using the 2100 Bioanalyzer or Fragment Analyzer followed by library quantification (Illumina library quantification kit). Sequencing was carried out on a HiSeq1500 or HiSeq2500 platform (Illumina). RNA-seq data shown in Fig. 2b–e, is from ribosome depleted RNA-seq and data shown in Figs 2a and 5 and Supplementary Figure S6 is from polyA-enriched RNA-seq.

### DNAse digestion of chromatin and Illumina sequencing

Livers were isolated from mice and nuclei were immediately purified using previously described protocol^54^ and resuspended in buffer (15 mM Tris-HCl pH 8.0, 15 mM NaCl, 60 mM KCl, 1 mM EDTA, 0.5 mM EGTA, 0.5 mM Spermidine and protease inhibitors) in a final concentration of 10 million nuclei per ml. DNase digestions were performed by adding 100 μl 10X digestion buffer (60 mM CaCl_2_ and 750 mM NaCl) containing 40 U or 60 U of DNase I (Sigma). Digestions were incubated for 3 min at 37 °C and reactions were terminated by addition of one volume of stop buffer (50 mM Tris-HCl, 100 mM NaCl, 0.1% SDS, 100 mM EDTA and 50 μg/ml Proteinase K (Ambion)). Digested chromatin was incubated at 55 °C for 2 h and stored at 4 °C until further use. DNase I digestion efficiency was evaluated by quantitative PCR^55^ and samples with optimal digestion efficiency were incubated with 90 μg/ml RNase A (Sigma) for 30 min at 37 °C before 50–500 bp DNA fragments were purified using ultracentrifugation. DNA was subsequently phenol/chloroform purified and ethanol precipitated. DNase-seq libraries were constructed according to manufacturer´s (Illumina) instructions as described previously^56^. Sequencing was carried out on a HiSeq1500 or HiSeq2500 platform (Illumina).

### Chromatin immunoprecipitation and Illumina sequencing

Liver tissue (~60 mg) was homogenized in PBS containing formaldehyde (1%), incubated for 10 min with rotation at RT, followed by quenching with glycine (0.125 M) for 10 min at RT. Homogenized liver tissue was washed two times in cold PBS, resuspended in 1 ml lysis buffer (0.1% SDS, 1% Triton X-100, 150 mM NaCl and 20 mM HEPES pH 7.6) and subsequently sonicated using a Biorupter (Diagenode). Chromatin was precleared using protein agarose A/G beads (Santa Cruz, sc-2003) for 30 min, rotating at 4 °C followed by immunoprecipitation using H3K27Ac antibody (Abcam, Ab4729) and protein agarose A/G beads ON at 4 °C. Precipitated chromatin was washed extensively, chromatin was eluted (1% SDS, 1 M NaHCO_3_) by 20 min rotation at RT and decrosslinked at 65 °C ON. DNA was purified using phenol/chloroform and precipitated with ethanol. DNA concentration was determined using Quant-iT PicoGreen dsDNA assay kit (ThermoScientific, P11496). ChIP-seq libraries were constructed according to manufacturer’s (Illumina) instructions as described previously^56^. Sequencing was carried out on a HiSeq1500 platform (Illumina).

### Analysis of RNA, ChIP and DNase sequencing

*RNA-seq:* Data was aligned to the mouse (mm9) reference genome using TopHat v.2.0.8 software^57^ or STAR^58^. Differential mRNA expression was analyzed by DESeq2 or iRNA using three biological replicates^22^,^23^. *DNase-seq:* Data was aligned to mm9 using STAR^58^ or Bowtie^59^. Replicate concordant DNase accessible regions were identified using HOMER at a FDR threshold of 0.001 and tag density threshold of 35^27^. Differentially DNase accessible regions were analyzed using DESeq2 from two biological replicates. *ChIP-seq:* Data was aligned to mm9 using STAR. Differential H3K27Ac was analyzed using DESeq2 from three biological replicates. Enriched DNA motifs at accessible regions were identified using HOMER^27^ and heatmaps of RNA-seq and ChIP-seq data were generated using MeV^60^.

## Additional Information

**Accession codes**: The Illumina sequencing data generated in this study has been deposited in the Gene Expression Omnibus (GEO) hosted by the National Center for Biotechnology Information under accession GSE88818 (DNase-seq and Ribozero based RNA-seq) and GSE87565 (H3K27Ac ChIP-seq and PolyA-based RNA-seq).

**How to cite this article**: Siersbæk, M. *et al*. High fat diet-induced changes of mouse hepatic transcription and enhancer activity can be reversed by subsequent weight loss. *Sci. Rep.* **7**, 40220; doi: 10.1038/srep40220 (2017).

**Publisher's note:** Springer Nature remains neutral with regard to jurisdictional claims in published maps and institutional affiliations.

## Supplementary Material

Supplementary Figures and Tables
