# Supplementary material for: High fat diet-induced changes of mouse hepatic transcription and enhancer activity can be reversed by subsequent weight loss
Source: Sci Rep. 2017 Jan 10;7:40220. doi: 10.1038/srep40220 (PMC5223143; doi:10.1038/srep40220)
Supplement: Supplementary Figures and Tables [file srep40220-s1.pdf]

Supplementary information

High fat diet-induced changes of mouse hepatic transcription and enhancer activity can be reversed by subsequent weight loss

Majken Siersbæk, Lyuba Varticovski, Shutong Yang, Songjoon Baek, Ronni Nielsen, Susanne Mandrup, Gordon L. Hager, Jay H. Chung, Lars Grøntved

Supplementary table S1. Summary of Illumina sequencing. Sequencing data was aligned to mm9 using TopHat (RNA-seq [Ribozero]), Bowtie (DNase-seq) or STAR (RNA-seq [PolyA], DNase-seq and ChIP-seq). RNA-seq, PolyA represents RNA-seq data from polyA-enriched RNA. RNA-seq, Ribozero represents RNA-seq data produced from RNA-depleted ribosomal RNA.

Sample ID	Method	Uniquely aligned reads
**Chow, rep1**	RNA-seq, PolyA	11720171
**Chow, rep2**	RNA-seq, PolyA	11783129
**Chow, rep3**	RNA-seq, PolyA	10588812
**HFD, rep1**	RNA-seq, PolyA	10369184
**HFD, rep2**	RNA-seq, PolyA	10279207
**HFD, rep3**	RNA-seq, PolyA	7455851
**HFD-chow, rep1**	RNA-seq, PolyA	11438832
**HFD-chow, rep2**	RNA-seq, PolyA	11224943
**HFD-chow, rep3**	RNA-seq, PolyA	14074351
**Chow-chow, rep1**	RNA-seq, PolyA	11927319
**Chow-chow, rep2**	RNA-seq, PolyA	11897382
**Chow-chow, rep3**	RNA-seq, PolyA	9777484
**Chow, rep1**	RNA-seq, Ribozero	49261677
**Chow, rep2**	RNA-seq, Ribozero	50060785
**Chow, rep3**	RNA-seq, Ribozero	51125127
**HFD, rep1**	RNA-seq, Ribozero	55636861
**HFD, rep2**	RNA-seq, Ribozero	43146179
**HFD, rep3**	RNA-seq, Ribozero	39882859
**Chow, rep1**	DNase-seq 40U	11751937
**Chow, rep2**	DNase-seq 40U	13081505
**HFD, rep1**	DNase-seq 40U	11496699
**HFD, rep2**	DNase-seq 40U	13026425
**Chow, rep1**	DNase-seq 60U	30797401
**Chow, rep2**	DNase-seq 60U	28945099
**HFD, rep1**	DNase-seq 60U	35524039
**HFD, rep2**	DNase-seq 60U	33956408
**Chow, rep1**	H3K27Ac ChIP-seq	15077420
**Chow, rep2**	H3K27Ac ChIP-seq	14388057
**Chow, rep3**	H3K27Ac ChIP-seq	12629852
**HFD, rep1**	H3K27Ac ChIP-seq	10621099
**HFD, rep2**	H3K27Ac ChIP-seq	13060025
**HFD, rep3**	H3K27Ac ChIP-seq	7298490
**HFD-chow, rep1**	H3K27Ac ChIP-seq	11659004
**HFD-chow, rep2**	H3K27Ac ChIP-seq	12156868
**HFD-chow, rep3**	H3K27Ac ChIP-seq	12057716
**Chow-chow, rep1**	H3K27Ac ChIP-seq	11053602
**Chow-chow, rep2**	H3K27Ac ChIP-seq	11411917
**Chow-chow, rep3**	H3K27Ac ChIP-seq	12211452

Supplementary table S2. Gene ontology analysis using PANTHER. Overrepresentation test using Bonferroni correction for multiple testing.

PANTHER GO-Slim Biological Process	Fold Enrichment	P-value
**Lipid metabolic process (GO:0006629)**	5.01	1.08E-14
**Steroid metabolic process (GO:0008202)**	7.79	2.35E-10
**Fatty acid metabolic process (GO:0006631)**	6.91	2.15E-09
**Sensory perception of chemical stimulus (GO:0007606)**	< 0.2	9.21E-05
**Cholesterol metabolic process (GO:0008203)**	7.61	9.55E-04
**Metabolic process (GO:0008152)**	1.39	1.18E-03
**Sensory perception of smell (GO:0007608)**	< 0.2	4.06E-03
**Primary metabolic process (GO:0044238)**	1.39	1.21E-02
**Neurological system process (GO:0050877)**	0.33	1.78E-02
**Sensory perception (GO:0007600)**	0.23	1.94E-02
**Cellular amino acid metabolic process (GO:0006520)**	3.45	5.92E-02
**Gluconeogenesis (GO:0006094)**	13.22	6.51E-02
**Anion transport (GO:0006820)**	2.97	5.74E-01

Supplementary table S3. Reactome curated pathway analysis.

Reactome pathway analysis	FDR
**Activation of gene expression by SREBF (SREBP)**	6.18E-08
**Regulation of cholesterol biosynthesis by SREBP (SREBF)**	9.27E-07
**Circadian Clock**	0.001020081
**Fatty acid, triacylglycerol, and ketone body metabolism**	0.001020081
**Unwinding of DNA**	0.017989684
**Fatty Acyl-CoA Biosynthesis**	0.017989684
**Mitotic G1-G1/S phases**	0.018982142
**ChREBP activates metabolic gene expression**	0.034287085
**Triglyceride Biosynthesis**	0.037763381
**PPARA activates gene expression**	0.040182535

Supplementary figure S4

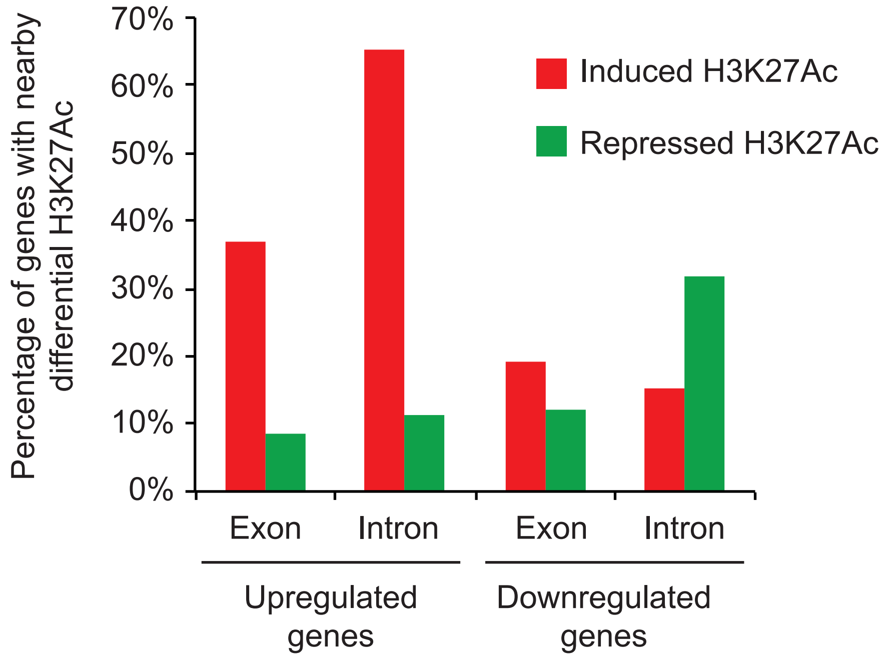

Supplementary figure S4. Frequency of HFD-induced up- and downregulated genes with nearby accessible regions that are differentially acetylated at H3K27 by HFD. Induced H3K27Ac is marked green and reduced H3K27Ac is marked red. Accessible regions within 100kb of the transcriptional start sites were selected for analysis. Differential gene expression was calculated using in intron or exon reads.

Supplementary figure S5

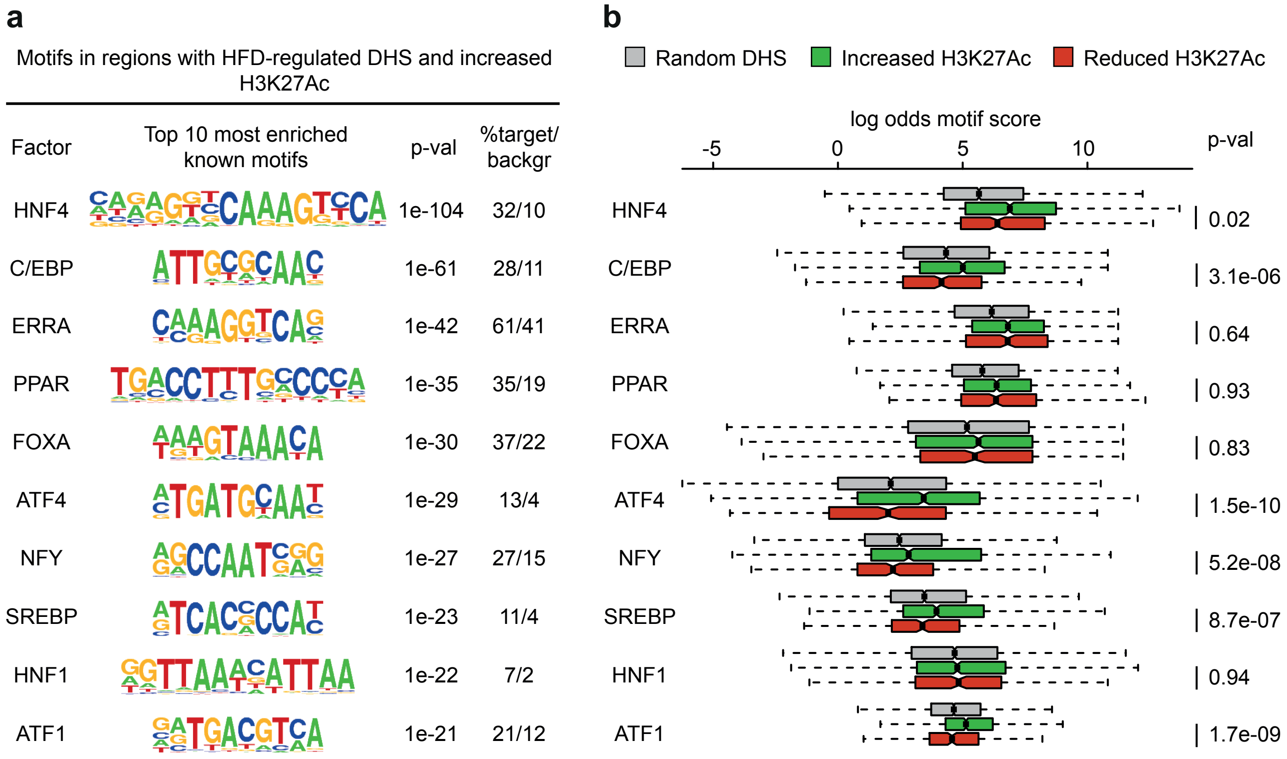

Supplementary figure S5. DNA motif analysis of HFD-regulated enhancers. **a)** DNA-sequence at DNase accessible regions with differentially regulated H3K27Ac by HFD was extracted and subjected to enriched motif analysis by HOMER. Top 10 of the most enriched known motifs. **b)** Motif strength of the most enriched motifs curated by HOMER at regions with either increased (green) or decreased (red) H3K27Ac or randomly selected DNase accessible regions (grey). Statistical test was performed by Wilcoxon signed-rank test.

Supplementary figure S6

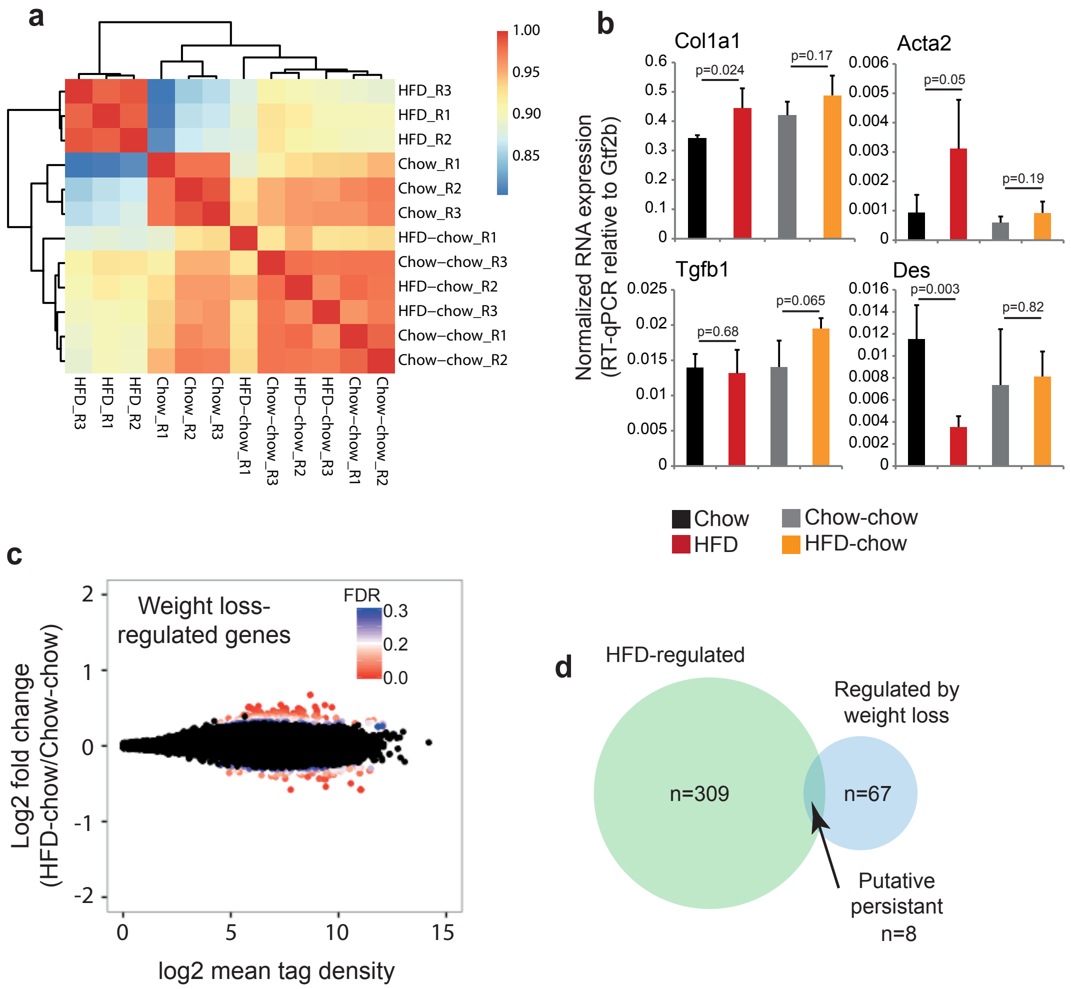

Supplementary figure S6. a) Pairwise corralation analysis (Pearson) of RNA-seq replicates from Chow, HFD, HFD-chow and Chow-chow feeding regimens. RNA-seq data was generated from polyA-enriched RNA. Pairwise correlation analysis was clusted using eucledian distance computation. Heatmap shows Pearson correlation coefficient. **b)** Expression of genes associated with hepatic fibrosis measured by RT-qPCR (n=3-4). Statistical test was performed by two-tailed t-test. Error bars represent SEM. **c)** Differential RNA expression (RNA-seq) in livers isolated from Chow-chow compared to HFD-chow treated mice. DESeq2 was used to compute FDR. FDR>0.3 is colored black, while FDR<0.3 is colored according to the heatmap. Red represents most significantly regulated genes. **d)** Unique and commen regulated genes by HFD and genes regulated as a result of weight loss. Differentially regulated genes are scored at FDR<0.05 computed by DESeq2. Commen regulated genes (8 in total) represents putative persistant regulated genes as a result of HFD.

Supplementary figure S7

a

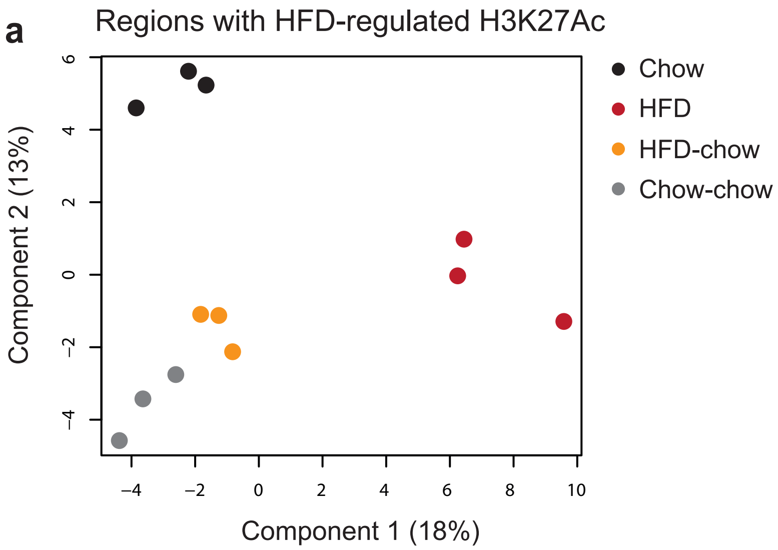

b

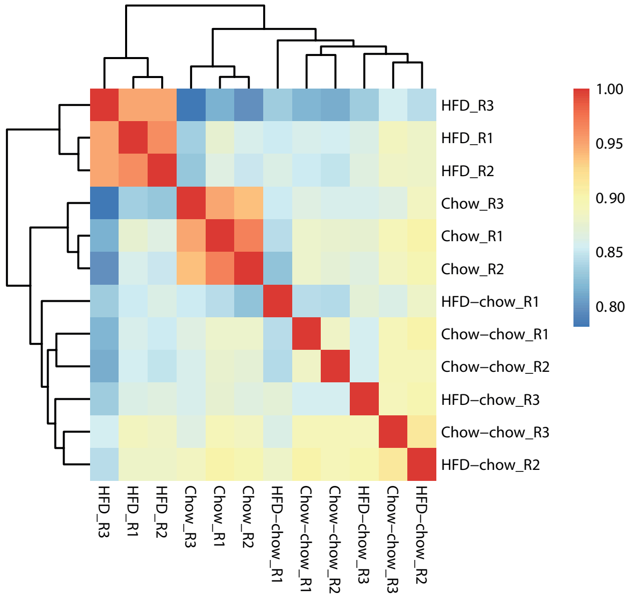

Supplementary figure S7. a) Principle component analysis and **b)** Pearson pairwise correlation analysis of H3K27Ac ChIP-seq replicates. Analysis was performed on regions demonstrating differential H3K27Ac in response to HFD ($p<0.05$). Pairwise correlation analysis was clusted using eucledian distance computation. Heatmap shows Pearson correlation coefficient.
